# Efficacy of Prednisolone in Generated Myotubes Derived From Fibroblasts of Duchenne Muscular Dystrophy Patients

**DOI:** 10.3389/fphar.2018.01402

**Published:** 2018-12-03

**Authors:** Tsubasa Kameyama, Kazuki Ohuchi, Michinori Funato, Shiori Ando, Satoshi Inagaki, Arisu Sato, Junko Seki, Chizuru Kawase, Kazuhiro Tsuruma, Ichizo Nishino, Shinsuke Nakamura, Masamitsu Shimazawa, Takashi Saito, Shin’ichi Takeda, Hideo Kaneko, Hideaki Hara

**Affiliations:** ^1^Molecular Pharmacology, Department of Biofunctional Evaluation, Gifu Pharmaceutical University, Gifu, Japan; ^2^Department of Clinical Research, National Hospital Organization, Nagara Medical Center, Gifu, Japan; ^3^Department of Molecular Therapy, National Institute of Neuroscience, National Center of Neurology and Psychiatry, Kodaira, Japan

**Keywords:** duchenne muscular dystrophy, prednisolone, utrophin, laminin, MMP-2

## Abstract

Duchenne muscular dystrophy (DMD) is a recessive X-linked form of muscular dystrophy characterized by progressive muscle degeneration. This disease is caused by the mutation or deletion of the dystrophin gene. Currently, there are no effective treatments and glucocorticoid administration is a standard care for DMD. However, the mechanism underlying prednisolone effects, which leads to increased walking, as well as decreased muscle wastage, is poorly understood. Our purpose in this study is to investigate the mechanisms of the efficacy of prednisolone for this disease. We converted fibroblasts of normal human cell line and a DMD patient sample to myotubes by MyoD transduction using a retroviral vector. In myotubes from the MyoD-transduced fibroblasts of the DMD patient, the myotube area was decreased and its apoptosis was increased. Furthermore, we confirmed that prednisolone could rescue these pathologies. Prednisolone increased the expression of not utrophin but laminin by down-regulation of *MMP-2* mRNA. These results suggest that the up-regulation of laminin may be one of the mechanisms of the efficacy of prednisolone for DMD.

## Introduction

Duchenne muscular dystrophy is an X-linked recessive inherited disease characterized by progressive muscle weakness and wasting that affects approximately 1 in 5000 newborn human males (Cowan et al., 1980). It is one of the most severe and common muscular dystrophies, with onset between the ages of 3 and 4. As the disease progresses, movement is gradually lost, eventually leading to motor paralysis, cardiomyopathy, and respiratory disorders. This disease is caused by mutations in the gene coding for dystrophin, resulting in the absence of dystrophin protein (Hoffman et al., 1987). Dystrophin is a membrane-associated protein and is a vital part of the dystrophin-glycoprotein complex, which is a transmembrane linker between laminin, the extracellular matrix component, and the cytoskeleton in skeletal muscle fibers (City, 1993). The absence of dystrophin leads to sarcolemma instability. And, the instability increases the Ca^2+^ level in myotubes (Mongini et al., 1988; Williams et al., 1990) and elevated the level of calcium-dependent proteolysis (Alderton and Steinhardt, 2000), which results in muscle wasting. Moreover, as other downstream mechanisms of DMD, muscle necrosis and apoptosis, inflammation, and oxidative stress have also been studied before (Acharyya et al., 2007; Whitehead et al., 2010; Chen et al., 2012).

Although the molecular origins of this disease have been known for years as presented above (Monaco et al., 1986; Hoffman et al., 1987), there are still no curative treatments available for DMD. Currently, the read-through drug, Translarna, conditionally has been approved in the European Union (Bushby et al., 2014) and a 30-nucleotide phosphorodiamidate morpholino oligomer provided *DMD* exon 51 skipping, Eteplirsen, was also approved by the United States Food and Drug Administration in September 2016 (Mendell et al., 2016). In Japan, treatment using glucocorticoids is used, and prednisolone is the only drug approved clinically to lessen the progression of DMD. The efficacy of glucocorticoids treatment for DMD was first reported in 13 of 14 patients approximately four decades ago (Drachman et al., 1974). In the years since, many studies involving glucocorticoids treatment for DMD have been reported. These studies showed that long-term glucocorticoid treatment prolonged the age at loss of ambulation, and improved muscle strength and cardiopulmonary function in the patients with DMD (Biggar et al., 2006; Houde et al., 2008; Takeuchi et al., 2013). Although many studies showed the clinical efficacy of glucocorticoid treatment for DMD as pointed out above, the mechanism of glucocorticoid action in DMD is poorly understood.

Glucocorticoids including prednisolone is generally known as an anti-inflammatory agent; therefore, it has also been considered that the main effects of glucocorticoids for DMD are anti-inflammatory and immunosuppressive actions. However, it has been previously described that the efficacy of glucocorticoid treatment for DMD cannot be explained by these actions alone (Weller et al., 1991; Kissel et al., 1993). Currently, multiple mechanisms of glucocorticoids have been proposed using animal disease models and myotubes from model mouse, including up-regulating utrophin, reduction of reactive oxygen species (ROS) production, modulating Ca^2+^ handling, and stability of the connection between the cytosol and the extracellular matrix (Passaquin et al., 1995; Miura et al., 2008; Tamma et al., 2013; Bozzi et al., 2015). However, studies using animal disease models and myotubes from model mouse have limitations in effective treatment approaches. Therefore, further understanding the detailed mechanisms underlying glucocorticoid action in the dystrophic muscles, particularly using human disease model, can help to develop the treatment for DMD. In this study, our attempt is to elucidate the mechanism of action of the prednisolone treatment for DMD using a human *in vitro* DMD model.

Herein, we describe the pathologies of myotubes derived from fibroblasts of DMD patients and the mechanism of the prednisolone treatment for DMD. We established a human *in vitro* DMD model, and found the decrease of dystrophin protein, smaller muscle fiber, and increased apoptosis. In addition, we confirmed that the prednisolone could rescue these DMD pathologies, and we could elucidate that a mechanism of the efficacy of prednisolone might consist in up-regulation of laminin and inhibition of *MMP-2* mRNA.

## Materials and Methods

### Ethics Statement

The patient samples were collected and used with the approval of the Ethical Review Committee of the National Hospital Organization. Informed consent was obtained from our patient. This study was carried out in accordance with the recommendations of the provisions of the Ethical Guidelines for Clinical Studies of the Ministry of Health. All subjects gave written informed consent in accordance with the Declaration of Helsinki.

### Multiplex Ligation-Dependent Probe Amplification Analysis

The multiplex ligation-dependent probe amplification (MLPA) analysis was carried out by LSI Medience Corporation, Japan.

### DNA Sequencing

Genomic DNA was collected from peripheral blood samples of DMD patient (DMD02) and *DMD* gene was analyzed using Ion PGM next-generation sequencer (Thermo Fisher Scientific, Waltham, MA, United States).

### Human MyoD-Transduced Fibroblasts Culture

The normal human fibroblast cell line TIG-119 was obtained from the National Institutes of Biomedical Innovation, Health and Nutrition (Osaka, Japan) and the dermal fibroblasts derived from patients with a regular visit to the National Hospital Organization, Nagara Medical Center for DMD were generated in our laboratory. These fibroblasts were cultured in a growth medium containing Dulbecco’s modified Eagle medium (DMEM)/F12 1:1 (Thermo Fisher Scientific, Rockford, IL, United States), 10% fetal bovine serum (FBS; Thermo Fisher Scientific), and 500 U/mL penicillin/streptomycin (Thermo Fisher Scientific) and were maintained in 5% CO_2_ at 37°C and passaged every seven days. At the differentiation stage, MyoD-transduced fibroblasts were cultured in a differentiation medium containing DMEM/F12 1:1, 2% horse serum (HS), 500 U/mL penicillin/streptomycin, and 1% Insulin-Transferrin-Selenium Supplement (ITS; Thermo Fisher Scientific, Rockford).

### MyoD Transduction

In the present study, we obtained the retrovirus encoding human MyoD and GFP from the National Institute of Neuroscience, National Center of Neurology and Psychiatry and modified a previously reported protocol that produced a retroviral vector and transduced the MyoD gene using the retroviral vector (Miller and Buttimore, 1986; Morgenstem and Land, 1990; Saito et al., 2010). When the retroviral vector was being produced, the expression vector and a pVSV-G envelope vector were co-transfected into a GP2-293 packaging cell line using the standard manual using Xfect transfection reagent (Clontech Laboratories, Inc.). After 48 h incubation, the viral supernatant was collected and stored at -80°C. In the retroviral transduction stage, the fibroblasts were harvested at 70–80% confluence in a 10 cm dish, and the retroviral stock was diluted with the growth medium to obtain the desired multiplicity of infection. We added Polybrene (Sigma-Aldrich, St. Louis, MO, United States) to a final concentration of 8 μg/ml and added the virus-containing transduction medium to the fibroblasts. Forty eight hours after incubation in 5% CO_2_ at 32°C, the virus-containing transduction medium was replaced with fresh growth medium and the fibroblasts were incubated at 37°C for 48 h. The MyoD-transduced fibroblasts were seeded in Matrigel-coated 96 well plates (Becton, Dickinson and Company, New Jersey, United States) at density of 5 × 10^4^ cell/cm^2^. After confirmation of cell attachment, the culture medium was changed to the differentiation medium. We cultured the MyoD-transduced fibroblasts for 11 to 21 days to differentiate into myotubes. This medium was changed every 2 or 3 days.

### Murine Myoblasts Culture

The murine myoblast cell line, C2C12 was purchased from the RIKEN Cell Bank (Cell No. RBRC-RCB0987, Tsukuba, Japan). Myoblasts were cultured in growth medium consisting of DMEM, 10% FBS, 100 U/mL penicillin (Meiji Co., Ltd., Tokyo, Japan), and 100 μg/mL streptomycin (Meiji Co., Ltd.) at 37°C in of 5% CO_2_. Two Days after starting culture of C2C12 myoblasts, the medium was changed to differentiation medium (DM), which consists of DMEM, 2% HS, 100 U/mL penicillin, and 100 μg/mL streptomycin, from day 2 to day 6, inducing MHC-expressed myotubes. C2C12 myoblasts were treated with Lipopolysaccharide (LPS; Sigma Aldrich, St. Louis, MO, United States) at a concentration of 1 μg/mL for 1 h.

### Western Blot Analysis

Samples were lysed in RIPA buffer (Sigma-Aldrich, St. Louis, MO, United States) containing 1% protease inhibitor cocktail and 1% of the phosphatase inhibitor cocktails 2 and 3 (Sigma-Aldrich), and harvested. The lysates were centrifuged at 12,000 *g* for 15 min at 4°C. The protein concentration was measured with a BCA Protein Assay Kit (Thermo Fisher Scientific) with bovine serum albumin as a standard. An equal volume of protein sample and sample buffer was mixed, and the samples were boiled for 5 min at 100°C. The protein samples were separated by 5–20% SDS-PAGE gradient electrophoresis and then transferred to polyvinylidene difluoride membranes (Immobilon-P; Millipore, Bedford, MA, United States). The primary antibodies used for immunoblotting were anti utrophin (sc-33700, santa cruz, Dallas, TX, United States) rabbit anti-cleaved caspase-3 (#9664, Cell Signaling Technology, Danvers, MA, United States), mouse anti-α7 integrin (sc-81807, santa cruz, Dallas, TX, United States), rabbit anti-laminin (ab11575, Abcam, Cambridge, MA, United States), rabbit anti-p-Akt (#4508, Cell Signaling Technology, Danvers, MA, United States), rabbit anti-total-Akt (t-Akt; #9272, Cell Signaling Technology, Danvers, MA, United States) and mouse monoclonal anti-β-actin (A2228, Sigma-Aldrich). A horseradish peroxidase (HRP)-conjugated goat anti-rabbit antibody (#32460, Thermo Fisher Scientific) and an HRP-conjugated goat anti mouse antibody (#32430, Thermo Fisher Scientific) were used as secondary antibodies. Immunoreactive bands were made visible by Immunostar-LD (Wako) and a LAS-4000 luminescent image analyzer (Fuji Film Co., Ltd., Tokyo, Japan). β-actin was used as the loading control.

### Drug Assay

The myotubes derived from the MyoD-transduced fibroblasts were exposed to 0.1 μg/ml prednisolone (Wako, Osaka, Japan) dissolved in ethanol for 11–16 or for 16–21 days of our differentiation protocol by changing the medium every 3 days. On the other hand, control cells received ethanol mixed with differentiation media. Then, the treated cells were washed with phosphate buffered saline (PBS, Thermo Fisher Scientific) and collected for each assay.

### Immunocytochemistry

After washing the plated cells twice with PBS, they were fixed in 4% paraformaldehyde (Nacalai Tesque, Kyoto, Japan) for 20 min at 4°C and then washed again with PBS. The cells were then blocked against non-specific labeling with 5% donkey serum and 0.1% Triton X-100 (Nacalai Tesque) in PBS for 30 min at 4°C. The cells were washed twice with PBS, and incubated with primary antibodies overnight at 4°C. After washing the cells twice with 0.1% Triton X-100 in PBS, incubation with the appropriate secondary antibody-tagged fluorescent dye was performed for 60 min at room temperature. The cell nucleus was labeled with Hoechst 33342 (Thermo Fisher Scientific). The primary antibodies used were anti-dystrophin (NCL-Dys3, diluted 1:20, Novocastra, Newcastle upon Tyne, United Kingdom), anti-myosin heavy chain (MHC, diluted 1:100, Cell Signaling Technology), anti-MyoD (diluted 1:50, santa cruz) and anti-cleaved caspase-3 (diluted 1:200, Cell Signaling Technology). The secondary Alexa Fluor-labeled antibodies used included 594 donkey anti-rabbit, 594 donkey anti-mouse IgG (Thermo Fisher Scientific, dilution for all second antibodies was 1:1000).

The cell count was performed by taking the five images per well. We also measured the area of MHC positive cells using BIOREVO BZ-9000 (Keyence, Osaka, Japan) for studying the size of generated myotubes and measured the MHC-positive area per MHC positive cell number. MHC-positive area was calculated using hybrid cell count software (Keyence’s original algorithm) and MHC positive cell was counted using image-processing software (Image J ver. 1.33f; National Institutes of Health, Bethesoda, MD, United States).

### ROS Assay

To measure cellular ROS production, the fluorescence signal intensity of CellROX^®^ Deep Red Reagent (Thermo Fisher Scientific, Rockford, IL, United States) was measured. Treated MyoD-transduced fibroblasts and non-treated fibroblasts were incubated with CellROX^®^ Deep Red Reagent for 30 min at 37°C and washed with PBS. After that, the signal was measured using the software of BIOREVO BZ-9000.

### Quantitative Real-Time Reverse Transcription Polymerase Chain Reaction

The expression of *MMP-2* mRNA was measured by quantitative real-time RT-PCR analysis. Samples were collected from control individual and DMD patients (DMD01 and DMD02). Total RNA was isolated according to the manufacturer’s protocol for NeuroSpin RNA II (Takara BIO INC., Shiga, Japan). First-strand cDNA was synthesized from total RNA in a 20-μL reaction mixture using the PrimeSprict RT reagent Kit (Takara). Real-time RT-PCR was performed with a Thermal Cycler Dice Real Time System II (Takara) using SYBR Premix Ex Taq II (Takara). The PCR protocol consisted of a 30-s denaturation step at 95°C, followed by a two-step PCR comprising 5 s at 95°C and 30 s at 60°C, with 45 cycles for *MMP-2* and *GAPDH*. For *MMP-2*, the forward primer was 5^′^-ATAACCTGGATGCCGTCGT -3^′^ and the reverse primer was 5^′^-AGGCACCCTTGAAGAAGTAGC -3^′^. For *GAPDH*, the forward primer was 5^′^- GAGTCAACGGATTTGGTCGT -3^′^, and the reverse primer was 5^′^- GACAAGCTTCCCGTTCTCAG-3^′^. Quantitative real-time RT-PCR analysis was performed using a Thermal Cycler Dice Real Time System TP 800 (Takara).

### Statistical Analysis

Data are presented as the mean ± standard error of the mean (SEM). The statistical significance of the data was evaluated using the one or two-tailed Student *t*-test. A *p* < 0.05 indicated statistical significance.

## Results

### Conversion of Fibroblasts to Myotubes and Establishment of Human *in vitro* DMD Model

**FIGURE 1:**
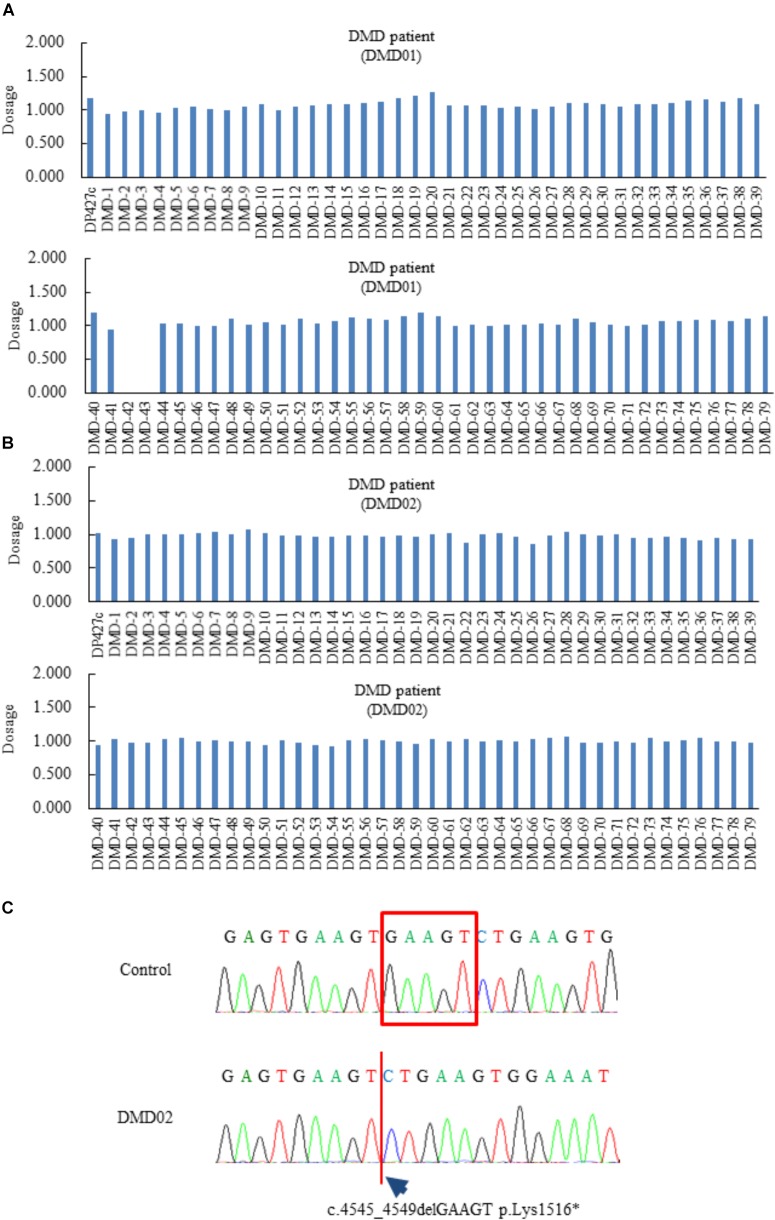
Genetic diagnosis of Duchenne muscular dystrophy (DMD) patients (DMD01 and 02) used in this study. **(A–C)** The Multiplex ligation-dependent probe amplification analysis of the DMD patients (DMD01 and 02) in this study. The exons 42 and 43 deletion were found in DMD01, but no deletion and duplication in DMD02. DMD02 showed c.4545_4549delGAAGT, p.Lys1516^∗^ in exon 33.

Initially, we generated dermal fibroblasts derived from a 56-year-old man (DMD01) and a 24-year-old man (DMD02) diagnosed as DMD. The patient (DMD01) had been previously diagnosed with a deletion of dystrophin exon 42 to 43 by MLPA analysis (Figure 1A). He showed difficulty walking at 17-years old and has used non-invasive ventilation with positive pressure delivered via a nasal mask at 32-years old. At present, he showed severe muscle weakness, wheelchair dependency, mild cardiac dysfunction, and respiratory failure on non-invasive ventilation with positive pressure. The patient (DMD02) was analyzed dystrophin gene by MLPA method at the age of 20 but the result indicated that the patient had no deletion or duplication in units of exons (Figure 1B). We analyzed all regions of dystrophin gene using next-generation sequencer and the patient (DMD02) and found that the patient had c.4545_4549delGAAGT p.Lys1516^∗^ in exon 33 (Figure 1C). His difficulty walking was observed at 7-years old and he showed significant heart failure with mitral regurgitation at 13-years old. Moreover, he has used non-invasive ventilation with positive pressure delivered via a nasal mask at 21-years old.

**FIGURE 2:**
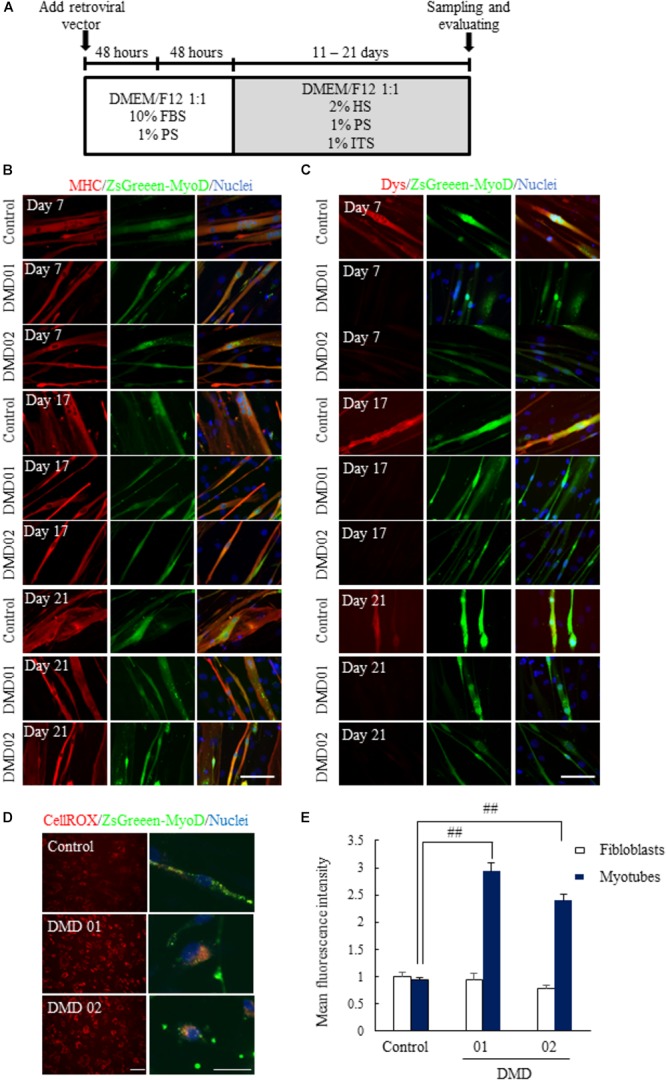
Conversion of fibroblasts to myotubes by MyoD transduction. **(A)** Schematic of myotubes differentiation from MyoD-transduced fibroblasts. **(B)** Immunostaining of skeletal muscle marker (myosin heavy chain, MHC) 11 to 21 days after myogenic differentiation. **(C)** Immunostaining of dystrophin 11 to 21 days after myogenic differentiation. The myotubes from MyoD-transduced fibroblasts of DMD patient resulted in the decrease of dystrophin protein. Scale bar = 50 μm. **(D,E)** ROS assay using CellROX^®^ deep red reagent to investigate whether ROS production was promoted or not. Data are shown as means ± SEM (*n* = 5). ^##^*p* < 0.01 vs. Control (Student’s *t*-test). Scale bar = 100 μm (left image), 50 μm (Right magnified image).

To establish human *in vitro* disease model derived from these DMD patients (DMD01 and 02), we transduced MyoD gene into the fibroblasts from these DMD patients using a previously reported protocol with modification (Figure 2A) and evaluated the expression of skeletal muscle marker and dystrophin. When we converted fibroblasts from both of normal human and DMD patients to myotubes by MyoD transduction, we firstly confirmed the expression of the MyoD in MyoD-transduced fibroblasts from both of normal human and DMD patients using anti-MyoD antibody (Supplementary Figure 1). In addition, we confirmed the expression of the MHC, skeletal muscle marker, in MyoD-transduced fibroblasts by immunostaining (Figure 2B). Next, we investigated the expression of dystrophin and confirmed the expression of dystrophin in MyoD-transduced fibroblasts of normal human, but the decrease of dystrophin in those of DMD (Figure 2C). These findings showed clearer results with culture time in myogenic differentiation medium (Figure 2C). Moreover, to identify that MyoD-transduced fibroblasts reflects the main hallmark of DMD, we measured ROS production using CellROX^®^ Deep Red Reagent. In our culture, ROS production was increased in myotubes not in fibroblasts (Figures 2D,E).

These results suggested that we could convert normal human and DMD fibroblasts to myotubes by MyoD transduction, and these myotubes reflected DMD pathology.

### Evaluating the DMD Pathologies Using Human *in vitro* DMD Model

**FIGURE 3:**
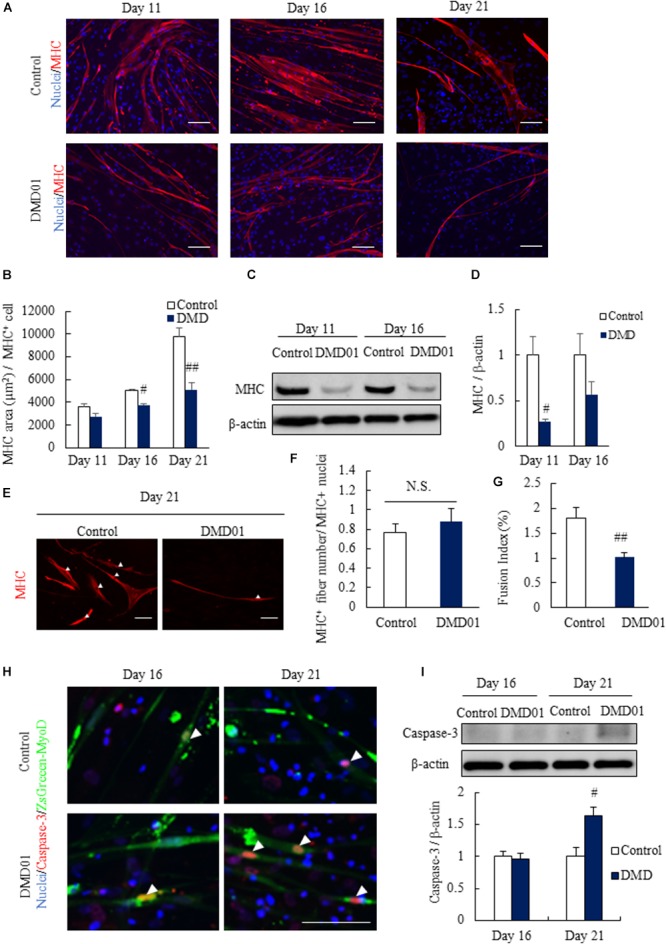
Comparison between myotues from MyoD-transduced fibroblasts of a normal human cell line and a DMD patient. **(A)** Comparison of myotubes from MyoD-transduced fibroblasts of a control individual and patient with DMD 11, 16, and 21 days after myogenic differentiation using skeletal muscle marker (myosin heavy chain, MHC). **(B)** Quantification of myotube area in MyoD-transduced fibroblasts of a control individual and a patient with DMD 11, 16, and 21 days after myogenic differentiation by analyzing MHC positive cells. Data are shown as means ± SEM (*n* = 3 or 5). ^##^*p* < 0.01 and ^#^*p* < 0.05 vs. Control (Student’s *t*-test). **(C,D)** Western blot analysis of MHC in a Ctrl individual and a DMD patient derived myotubes at 11 and 16 days. Data are shown as means ± SEM (*n* = 3). #*p* < 0.05 vs. Control (Student’s *t*-test). **(E,F)** Number of nuclei per MHC^+^ myotubes in Ctrl individual and a DMD patient at 21 days. Arrowheads indicate the nuclei. Data are shown as means ± SEM (*n* = 5). Scale bar = 100 μm. **(G)** Fusion index analysis in Ctrl individual and a DMD patient (DMD01). Data are shown as means ± SEM (*n* = 3). ^##^*p* < 0.01 vs. Control (Student’s *t*-test). **(H)** Comparison of death of myotubes from MyoD-transduced fibroblasts of a control individual and a patient with DMD 16 and 21 days after myogenic differentiation using apoptosis marker (cleaved caspase-3). Arrowheads indicate cleaved caspase-3 positive cells. Scale bar = 100 μm. **(I)** Western blot analysis of cleaved caspase-3 in myotubes from MyoD-transduced fibroblasts of a control individual and a DMD patient. Data are shown as means ± SEM (*n* = 3). ^##^*p* < 0.01 vs. Control and ^#^*p* < 0.05 vs. Control (Student’s *t*-test).

To evaluate the DMD pathologies, we evaluated the differences of myotubes from MyoD-transduced fibroblasts between normal human and DMD patient (DMD01). When MHC was expressed in both differentiated MyoD-transduced fibroblasts of normal human and DMD patient for 11 to 21 days by immunostaining, we found that the myotubes from MyoD-transduced fibroblasts of DMD patient showed a significantly reduction of MHC area compared with those of normal human on day 16 and 21 (Figures 3A,B). Although this finding was also suggested from day 11, it made clear from day 16 (Figures 3A,B). In addition, these differences of expression of MHC protein in myotubes from MyoD-transduced fibroblasts between normal human and DMD patient were also confirmed by western blot analysis (Figures 3C,D). Next, we investigated the number of nuclei per these myotubes and confirmed that it was not much difference in myotubes from between normal human and a DMD patient, suggesting that the reduction of MHC expression in DMD patient derived from MyoD-transduced fibroblasts was due to the impairment of the differentiation capacity (Figures 3E,F). Furthermore, to make this the data of MHC area reliable, fusion index analysis was performed. The data showed that fusion index in DMD patient derived from MyoD-transduced fibroblasts was significantly reduced, suggesting that fusion ability of DMD derived from MyoD-transduced fibroblasts was impaired (Figure 3G).

These results revealed that myotubes from MyoD-transduced fibroblasts of DMD patient showed smaller muscle fiber in a reflection of DMD pathology.

Furthermore, we investigated apoptosis and ROS production using myotubes from MyoD-transduced fibroblasts. In the apoptosis assay, we performed immunostaining by apoptosis marker cleaved caspase-3 and found that the expression of cleaved caspase-3 significantly increased in myotubes from MyoD-transduced fibroblasts of DMD patient sample differentiated for 21 days compared with those of normal human (Figure 3H). These results was quantitatively confirmed by western analysis (Figure 3I). Taken together, we could reveal an aspect of further DMD pathology that indicated reduction of myotube area from day 16 and increase in the level of apoptosis on day 21 in myotubes from MyoD-transduced fibroblasts of DMD patient using human *in vitro* DMD model.

### Efficacy of Prednisolone for DMD Using the Myotubes From DMD Patient Fibroblasts

**FIGURE 4:**
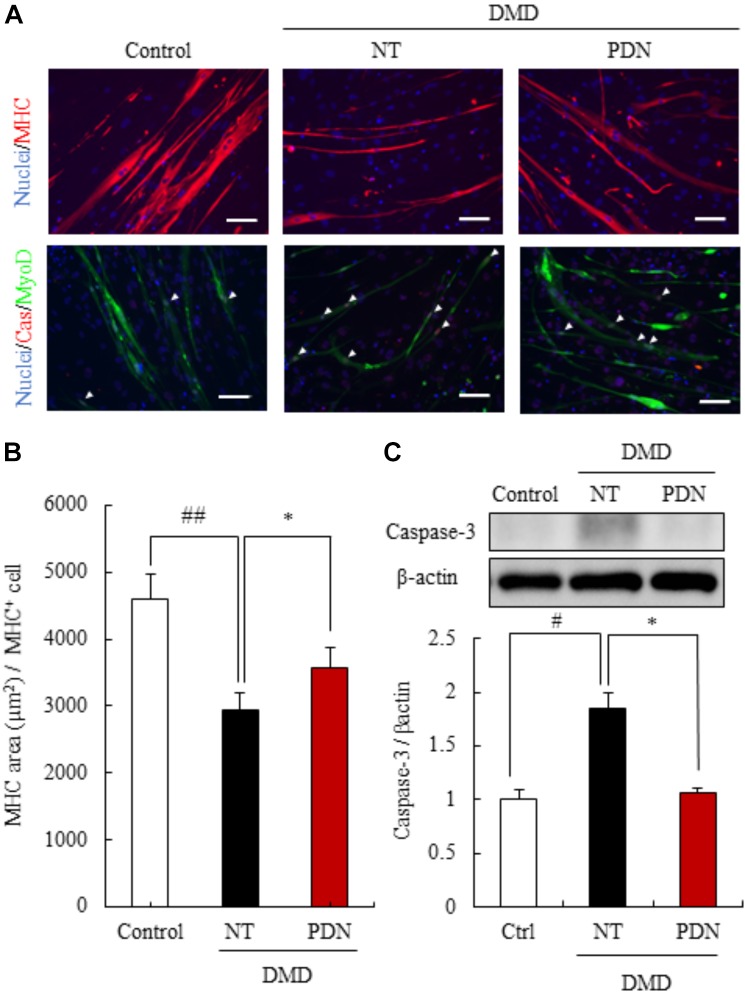
Efficacy of prednisolone in reversing DMD pathology. **(A)** Immunostaining using skeletal muscle marker (myosin heavy chain, MHC), and apoptosis marker (cleaved caspase-3, Cas). Prednisolone (PDN) restored decreased MHC area and increased apoptosis cells. Scale bars = 100 μm. **(B)** Quantification of the MHC area of myotubes from control individual, non-treated myotubes from DMD (NT), and prednisolone-treated myotubes from DMD (PDN). Data are shown as means ± SEM (*n* = 4). **(C)** Western blot analysis of cleaved caspase-3 in control individual (Ctrl), NT, and PDN. Data are shown as means ± SEM (*n* = 5). ^#^*p* < 0.05 vs. Ctrl and ^∗^*p* < 0.05 vs. NT (Student’s *t*-test), ^∗^*p* < 0.05 vs. NT (Student’s *t*-test).

To evaluate the efficacy of prednisolone for DMD pathologies, we treated the myotubes from fibroblasts of a milder DMD patient (DMD01) with prednisolone for ease of detection of prednisolone efficacy. We investigated whether prednisolone treatment could restore the area of myotubes, by employing immunostaining with MHC. When the myotubes from the MyoD-transduced fibroblasts of DMD patient were exposed to prednisolone for 11 to 16 days of our differentiation protocol, we found a significant increase in the area of myotubes from the MyoD-transduced DMD fibroblasts treated with prednisolone (Figures 4A,B). These results reveal that prednisolone could act the prevention of smaller muscle fiber in human DMD pathology.

Next, we studied whether prednisolone treatment for 16 to 21 days of our differentiation protocol could reduce apoptosis by immunostaining and western blot analysis of cleaved casepase-3. We found that the expression of cleaved caspase-3 in myotubes from the MyoD-transduced DMD fibroblasts treated with prednisolone significantly decreased compared with no treatment groups (Figure 4C). These results revealed that prednisolone was also able to act the prevention of apoptosis of muscle fiber in human DMD pathology.

Taken together, these results revealed that prednisolone could rescue DMD pathology in human *in vitro* DMD model and encouraged a part of the clinical efficacy of glucocorticoids treatment for DMD patient.

### Mechanisms of the Efficacy of Prednisolone for DMD Pathologies

**FIGURE 5:**
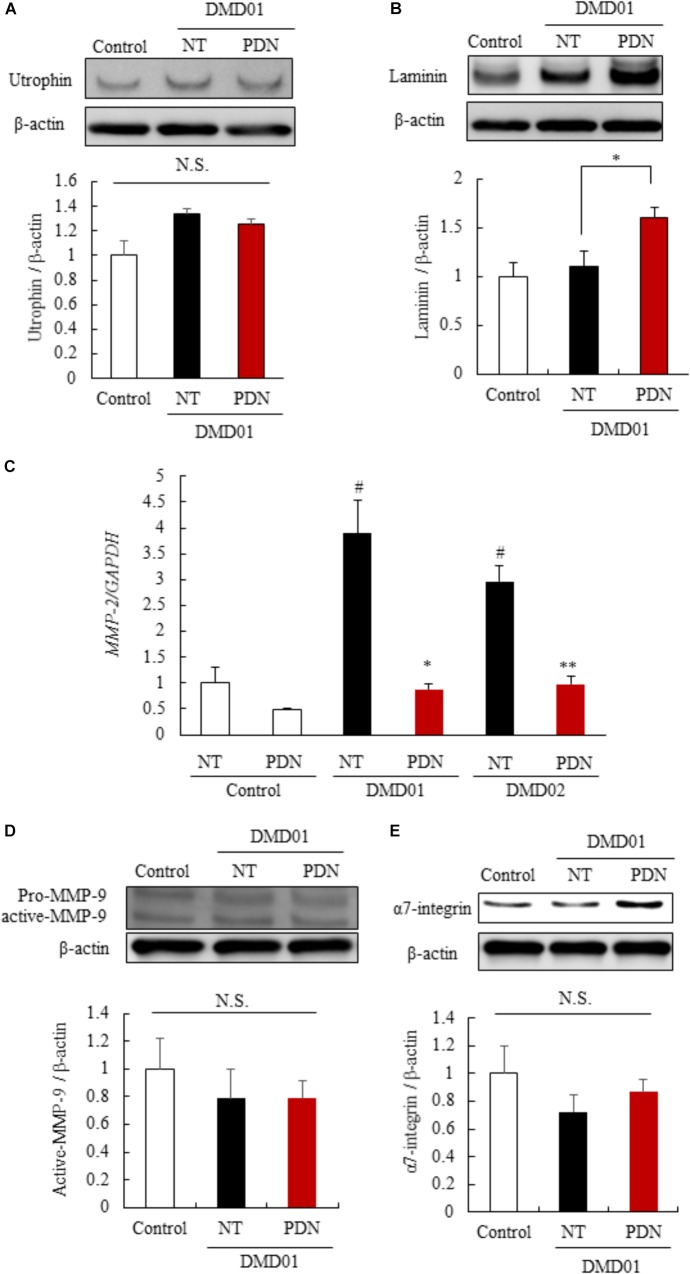
Mechanisms of the efficacy of prednisolone in DMD pathology. **(A)** Western blot analysis of utrophin in myotubes from control individual (Ctrl), NT, and PDN. **(B)** Western blot analysis of laminin in Ctrl, NT, and PDN. **(C)** Quantitative RT-PCR analysis of *MMP*-2 in Ctrl, NT, and PDN. **(D)** Western blot analysis of MMP-9 in Ctrl, NT, and PDN. **(E)** Western blot analysis of α7 integrin in Ctrl, NT, and PDN. Data in Figure are shown as means ± SEM (*n* = 3). ^#^*p* < 0.05 vs. Ctrl, ^∗∗^*p* < 0.01, ^∗^*p* < 0.05 vs. NT (Student’s *t*-test).

To identify the mechanisms of the efficacy of prednisolone in myotubes from the MyoD-transduced fibroblasts of DMD patient (DMD01), we investigated the reported mechanism using established human *in vitro* DMD model. We initially investigated the expression of the utrophin protein, which has a similar structure and function, using western blot analysis, as several groups have demonstrated that utrophin is up-regulated in response to glucocorticoid treatment (Tamma et al., 2013). When the myotubes from the MyoD-transduced fibroblasts were exposed to prednisolone for 11 to 16 days of our differentiation protocol, we found that there was no significant difference in expression of utrophin between no-treatment and prednisolone treated groups in human *in vitro* DMD model that we established (Figure 5A). Therefore, next we investigated the expression of laminin and α7 integrin, extracellular matrix components, using western blot analysis, as prednisolone treatment was reported to show a strong laminin and α7 integrin expression around the myofibers in mdx mice (Tamma et al., 2013; Wuebbles et al., 2013). Interestingly, we found that an increased expression of laminin in the prednisolone treated group compared with no treatment group (Figure 5B). Moreover, to identify the mechanism of up-regulation of laminin, we focused on the expression of MMP-2 and MMP-9 which specifically cleaved laminin (Giannelli et al., 1997; Ogura et al., 2014). We investigated the expression of MMP-2 and MMP-9 in myotubes from the MyoD-transduced fibroblasts of DMD patient using quantitative reverse transcription-polymerase chain reaction (RT-PCR) and western blot analysis. We found that prednisolone treatment down-regulated the expression of *MMP-2* mRNA in the myotubes from the MyoD-transduced fibroblasts of DMD patients (DMD01 and 02) (Figure 5C). In contrast, prednisolone treatment did not change the expression of MMP-9 (active form) (Figure 5D). This results revealed that prednisolone could increase the expression of laminin through down-regulation of *MMP-2* mRNA. However, prednisolone treatment did not change the expression of α7-integrin (Figure 5E). These results revealed that glucocorticoid treatment could increase the expression of laminin protein through the reduction of *MMP-2* mRNA, but could not change amount of utrophin and α7-integrin in myotubes from the MyoD-transduced fibroblasts of DMD patient. This finding may indicate that there is little or no ability of glucocorticoids treatment to up-regulate utrophin and α7-integrin in human.

Moreover, to confirm the change of laminin expression specifically in myotubes, we established the LPS-induced muscle atrophy model using C2C12, a mouse myoblast cell line. One of the hallmarks of DMD is the muscle inflammation, and dystrophin-depleted muscle releases IL-1β which induces inflammatory factors such as NF-κB (Weber et al., 2010). LPS also binds to Toll like receptor 4 (TLR4) and stimulates inflammatory factors such as NF-κB (Ono and Sakamoto, 2017), leading to the phenotype similar to DMD. Therefore, we investigated whether the expression of an extracellular matrix components such as laminin and α7-integrin by prednisolone treatment using the LPS-induced muscle atrophy model. C2C12 myotubes was treated with 2% horse serum from day 2 to day 7, LPS and prednisolone were added at day 6 and treated for 24 h (Supplementary Figure 2A). At first, we confirmed the expression of MHC was decreased in LPS-treated group like previous report (Supplementary Figure 2B; Ono and Sakamoto, 2017). Next, we found that the expression of laminin in the prednisolone treated group was also increased compared with no treatment group in the muscle atrophy model, although the expression of α7-integrin had no change (Supplementary Figures 2C–E). Moreover, the laminin restoration increases the expression of phosphorylated Akt (p-Akt) and promotes cell survival in lung cancer cell and muscle cell (Tsurutani et al., 2005; Marshall et al., 2012). Therefore, we measured the expression of cell survival signal related factor, p-Akt. In this model, the expression of phosphorylated Akt was increased (Supplementary Figures 2C,F), suggesting that prednisolone treatment promoted muscle cell survival by augmenting the expression of laminin, not α7-integrin.

## Discussion

In the present study, we generated the myotubes from normal human and DMD patient fibroblasts and established human *in vitro* DMD model. This DMD model using myotubes from fibroblasts of DMD patients revealed aspects of DMD pathologies, including the deletion of dystrophin exon 42 to 43 (Figures 1A,B), the decrease of dystrophin protein (Figure 2C), increased ROS production (Figures 2D,E), reduction of myotube area (Figures 3A–F), and an increase in the level of apoptosis (Figures 3G,H). Then, we showed that glucocorticoids that have been shown to improve DMD pathologies in clinical practice could restore these problem to near-normal state (Figure 4). As our report in this study, there have been some reports indicating the efficacy of glucocorticoids treatment for DMD through the use of animal disease models and *in vitro* model using myotubes of mouse (Passaquin et al., 1995; Miura et al., 2008; Tamma et al., 2013). However, there is few reports about the mechanisms of the efficacy of glucocorticoids for DMD, in particular using human *in vitro* DMD model. Therefore, we studied the mechanisms using a human *in vitro* DMD model established and showed that the up-regulation of laminin through the down-regulation of *MMP-2* mRNA was at least associated with the efficacy of prednisolone (Figures 5B,C).

Currently, there are still no curative agents for DMD. Many of current treatment approaches revealed therapeutic potential in the animal disease models such as the mdx mouse or dogs (Mann et al., 2001). Therefore, these lack of useful *in vitro* human DMD models may have contributed to delaying the development of effective treatment for this disease. The most common cell source for investigating human DMD *in vitro* is via muscle biopsy from patients with this disease (Blau et al., 1983; Delaporte et al., 1990). However, a muscle biopsy from patients with DMD is an invasive method, and the proliferation capacity of these cells is impaired (Blau et al., 1983; Delaporte et al., 1990). Recently, the studies of pathogenesis and drug evaluation using human induced pluripotent stem cells and myogenic conversion of fibroblasts by MyoD transduction have been reported (Saito et al., 2010; Shoji et al., 2015) as alternatives that can produce a large number of myotubes repeatedly. In this study, we were also able to evaluate the mechanism of the efficacy of prednisolone using a human *in vitro* DMD model but this model have some limitations for further accurate elucidation of mechanism. One is the respect that reprogramming efficiency is not high. It was difficult to identify whether the change of expression level in Western blotting is due to myotube cells or fibroblasts, in MyoD-transduced fibroblasts. To resolve the problem, we confirmed the reproducibility of the action of prednisolone using mouse muscle cell line, C2C12 in the Supplementary Figure 2. Therefore, the expression of laminin *in vitro* human DMD model may be increased in myotubes according to the data of C2C12. Another is the respect that the myotubes from MyoD-transduced fibroblasts are not mature muscle fibers (Shoji et al., 2015). Although we represented myotubes from MyoD-transduced fibroblasts as a human *in vitro* DMD model, it is important to improve this model by the cell sorter such as fluorescence activated cell sorting (FACS) for further detail analysis in future. Furthermore, it is thought that differences in drug response may depend on individuals or species, therefore more detailed analysis using samples from many patients with DMD is necessary.

Prednisolone is a corticosteroid drug that has anti-inflammatory and immunosuppressive effects. Long-term prednisolone treatment improved walking, muscle strength, and cardiopulmonary function in randomized controlled trials (Takeuchi et al., 2013). Although the clinical efficacy of prednisolone is well known as above, the mechanisms is still not understood and remain controversy. The ability of glucocorticoids treatment to up-regulate utrophin has recently been well known (Tamma et al., 2013). However, our study indicated little or no efficacy of prednisolone to utrophin in human DMD patient (DMD01) (Figure 5A). Moreover, the increased ROS production in muscle fibers caused by the dystrophin deletion is an important finding of DMD pathology (Figures 2D, E). In addition, Miyazaki et al. described that muscle fiber in mdx mice might be regenerated by not MMP-2 but rather MMP-9 (Miyazaki et al., 2011). We found that *MMP-2* mRNA expression was increased by prednisolone whereas MMP-9 expression did not change in evaluating the mechanism of up-regulation of laminin by prednisolone (Figures 5C,D). These data indicate that these differences might depend on individuals or species. For instance, we mainly studied an atypical DMD patient in a 56-year-old with the deletion of dystrophin exon 42 and 43 whose mutation is relatively rare (Koenig et al., 1989) and who revealed the mild phenotype similar to BMD. Therefore more detailed analysis using samples from many patients with DMD is necessary concerning expression of utrophin, ROS production, the role of MMP in regeneration of muscle fiber, and others by prednisolone treatment.

The current treatment approaches can be categorized into three major groups, including genetic therapies using adeno-associated virus and antisense oligonucleotides (Wang et al., 2000; Mann et al., 2001), up-regulating surrogate proteins (Tinsley et al., 2011), targeting the downstream disease mechanisms. In this study, prednisolone improved the DMD pathologies by not up-regulation of utrophin protein but both up-regulation of laminin protein through the down-regulation of *MMP-2* mRNA and promotion of cell survival such as inhibition of the expression of cleaved caspase 3 and up-regulation of p-Akt expression (Figures 4, 5A–C and Supplementary Figure 2). Laminin treatment is reported to protect muscle from excised-induced damage in mdx mouse (Rooney et al., 2009). The main efficacy of prednisolone for human DMD patients may be muscle protection by increased laminin protein.

In conclusion, glucocorticoids treatment for DMD might be not able to enhance the expression of utrophin protein in human DMD. However, it contributed to an increased expression of laminin protein through the down-regulation of *MMP-2* mRNA. These clues must be able to provide further effective treatment for DMD.

## Author Contributions

TK, KO, MF, KT, SN, MS, HK, and HH conceived and designed the experiments. TK, KO, SA, SI, AS, JS, and CK performed the analysis and the experiments. TS and ST provided the study materials. IN diagnosed DMD patient (DMD02). TK, KO, MF, HK, and HH wrote the paper. All authors reviewed the manuscript.

## Conflict of Interest Statement

The authors declare that the research was conducted in the absence of any commercial or financial relationships that could be construed as a potential conflict of interest.
