# Supplementary figures and images for: Efficacy of Prednisolone in Generated Myotubes Derived From Fibroblasts of Duchenne Muscular Dystrophy Patients
Source: Front Pharmacol. 2018 Dec 3;9:1402. doi: 10.3389/fphar.2018.01402 (PMC6287205; doi:10.3389/fphar.2018.01402)

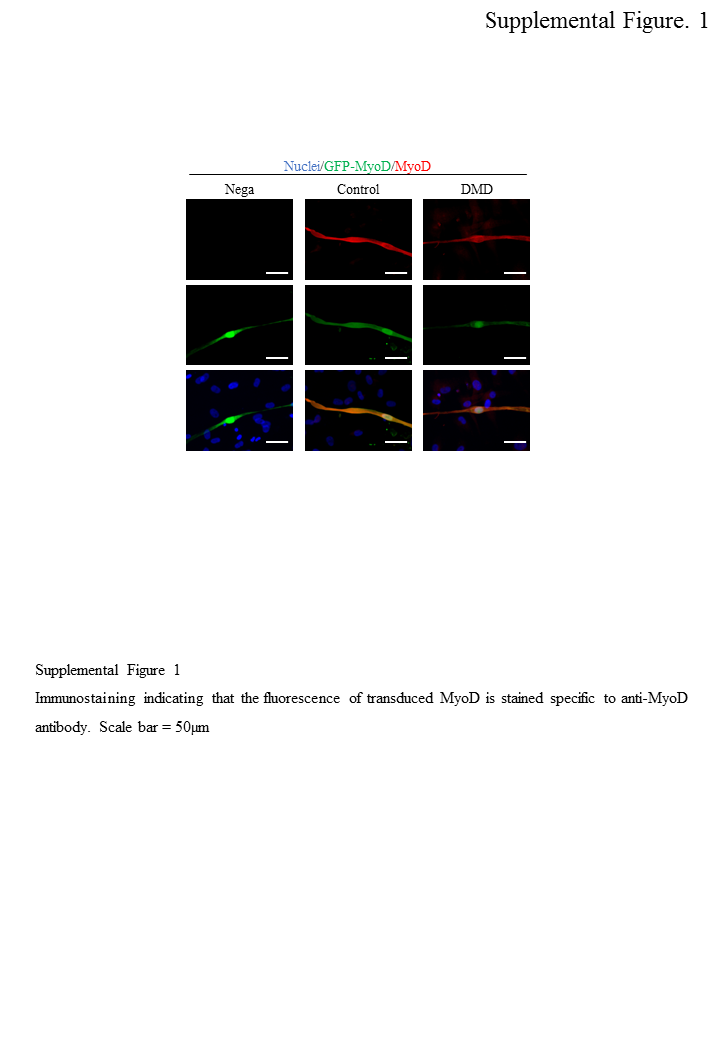

Supplement: Supplementary file 1 [file Image_1.TIF]

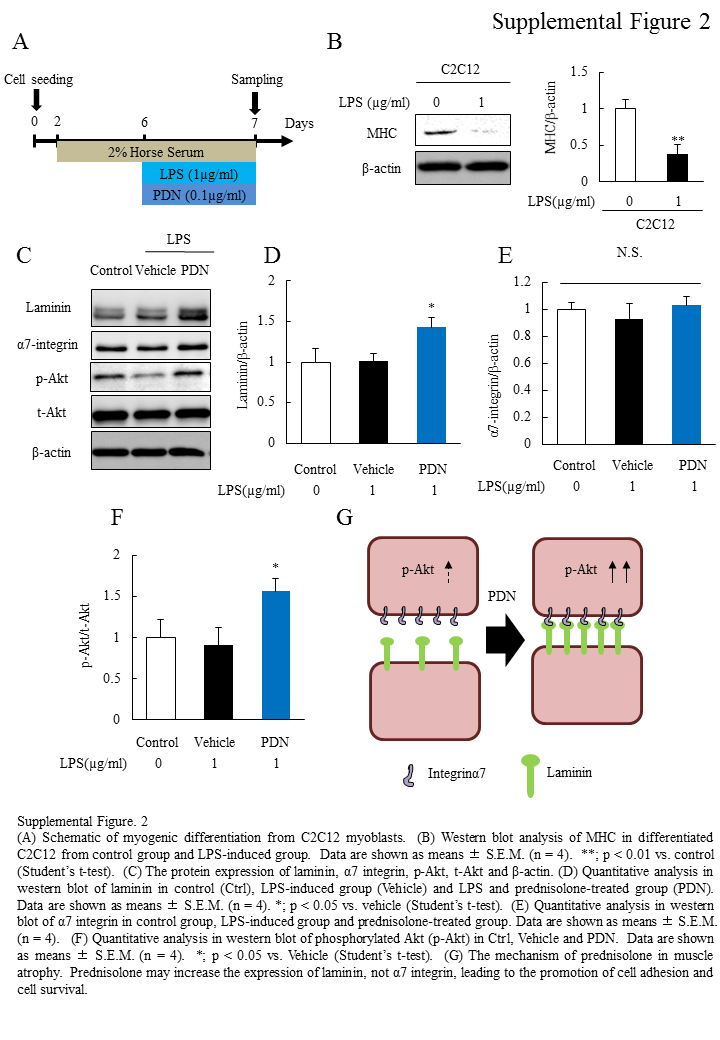

Supplement: Supplementary file 2 [file Image_2.TIF]
